# Confluent and Reticulated Papillomatosis of Gougerot and Carteaud: A Case Report and Review of the Literature

**DOI:** 10.7759/cureus.24557

**Published:** 2022-04-28

**Authors:** Tasneem A Banjar, Rahaf A Abdulwahab, Khalid A Al Hawsawi

**Affiliations:** 1 College of Medicine, Umm Al-Qura University, Makkah, SAU; 2 Dermatology, King Abdulaziz Hospital, Makkah, SAU

**Keywords:** confluent and reticulated papillomatosis, dermatology case report, gougerot and carteaud, pediatric case, rare presentation, generalized lesions

## Abstract

Confluent and reticulated papillomatosis (CARP) of Gougerot and Carteaud is a rare chronic disease with exacerbation and remissions typically affecting young people. Classic clinical characteristics include asymptomatic scaly hyperpigmented macules, patches, and papules in the trunk's confluent and reticular pattern.

A 12-year-old girl, otherwise healthy, presented with itchy, persistent skin lesions all over her body for one year. Skin examination revealed generalized scaly brownish patches, thin papules, and plaques all over her body, including her face, neck, middle of the chest, abdomen, back, upper extremities, elbows, lower extremities, and knees. Wood's lamp examination of her skin lesions was unrevealing. Skin biopsy showed papillomatosis, hyperkeratosis, acanthosis, and hypergranulosis. The dermis showed perivascular inflammatory cellular infiltrate. Based on the above clinicopathological findings, the patient was diagnosed with CARP. In our case, a generalized form was reported. CARP is diagnosed based on clinical and histopathological features. Oral antibiotics are the cornerstone of treatment. Our patient responded well to oral minocycline 85 mg one tablet daily, tacrolimus 0.1% ointment twice daily, and selenium sulfide shampoo twice weekly for two months.

The classic clinical characteristics of CARP include asymptomatic scaly hyperpigmented macules, patches, and papules in a confluent and reticular pattern on the trunk. A generalized form, as in our case, has been reported. CARP is diagnosed based on clinical and histopathological features. Oral antibiotics are the cornerstone of treatment.

## Introduction

Confluent and reticulated papillomatosis (CARP) of Gougerot and Carteaud is a rare chronic disease, consisting of exacerbations and remissions, and it typically affects young people. It is characterized by asymptomatic scaly, hyperpigmented papules and plaques that are reticulated at the periphery and confluent in the center. A hypopigmented variant of CARP was previously described [1]. This variant typically affects the intermammary region, epigastric area, and upper back, and less commonly, the neck, axillae, shoulders, and face. The precise underlying cause has not been determined yet. Abnormal host reaction to *Pityrosporum* organisms or bacteria, hyperinsulinemia, insulin resistance, Cushing disease, menstrual irregularities, thyroid disease, pituitary dysfunction, hirsutism or hypertrichosis, obesity, acanthosis nigricans, ultraviolet light exposure, amyloidosis, and the disorder of keratinization with overexpression of keratin-16 have been suggested to play a role in the development of the disease [2]. Pregnant women or individuals who lose weight frequently experience CARP remission. Familial cases of the disease have been reported, but the familial occurrence is typically sporadic. This condition is quite common among adolescents and young adults with blacks suffering from the condition twice as much as whites. According to previous studies, some studies noted a male predominance while others noted a female predominance [2,3]. Treatment of the disease includes topical and systemic treatments. Systemic treatment includes minocycline, doxycycline, antifungals, retinoids (isotretinoin, acitretin), oral contraceptives, and/or phototherapy [2]. Topical treatment includes lactic acid, selenium sulfide shampoo, antifungals, mupirocin, retinoids, salicylic acid, urea, tacrolimus, and/or vitamin D analogs. A case of CARP with a generalized distribution is presented in this case report.

## Case presentation

**Figure 1:**
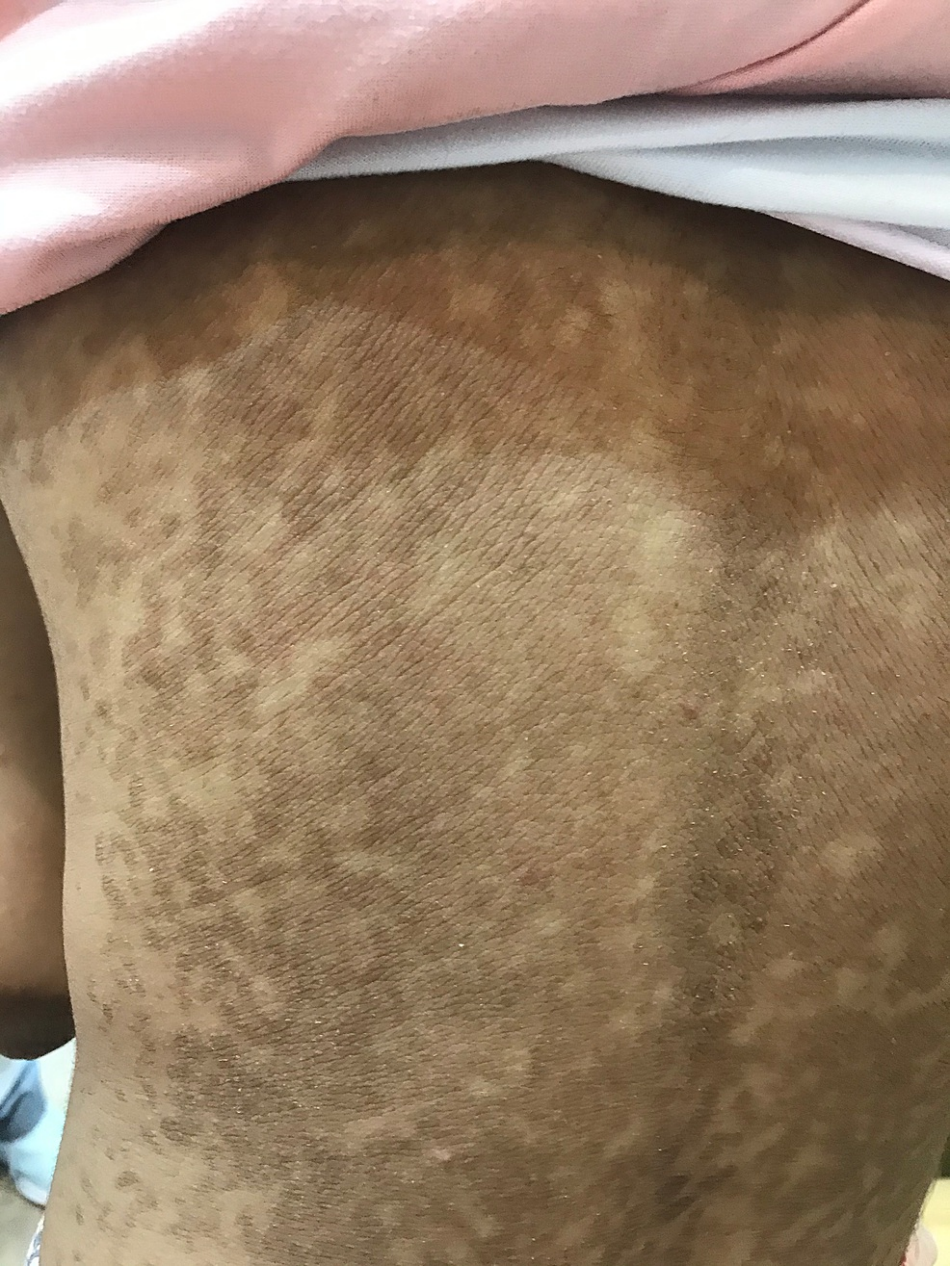
Reticulated hyperpigmented patches and plaques over the back of the patient.

**Figure 2:**
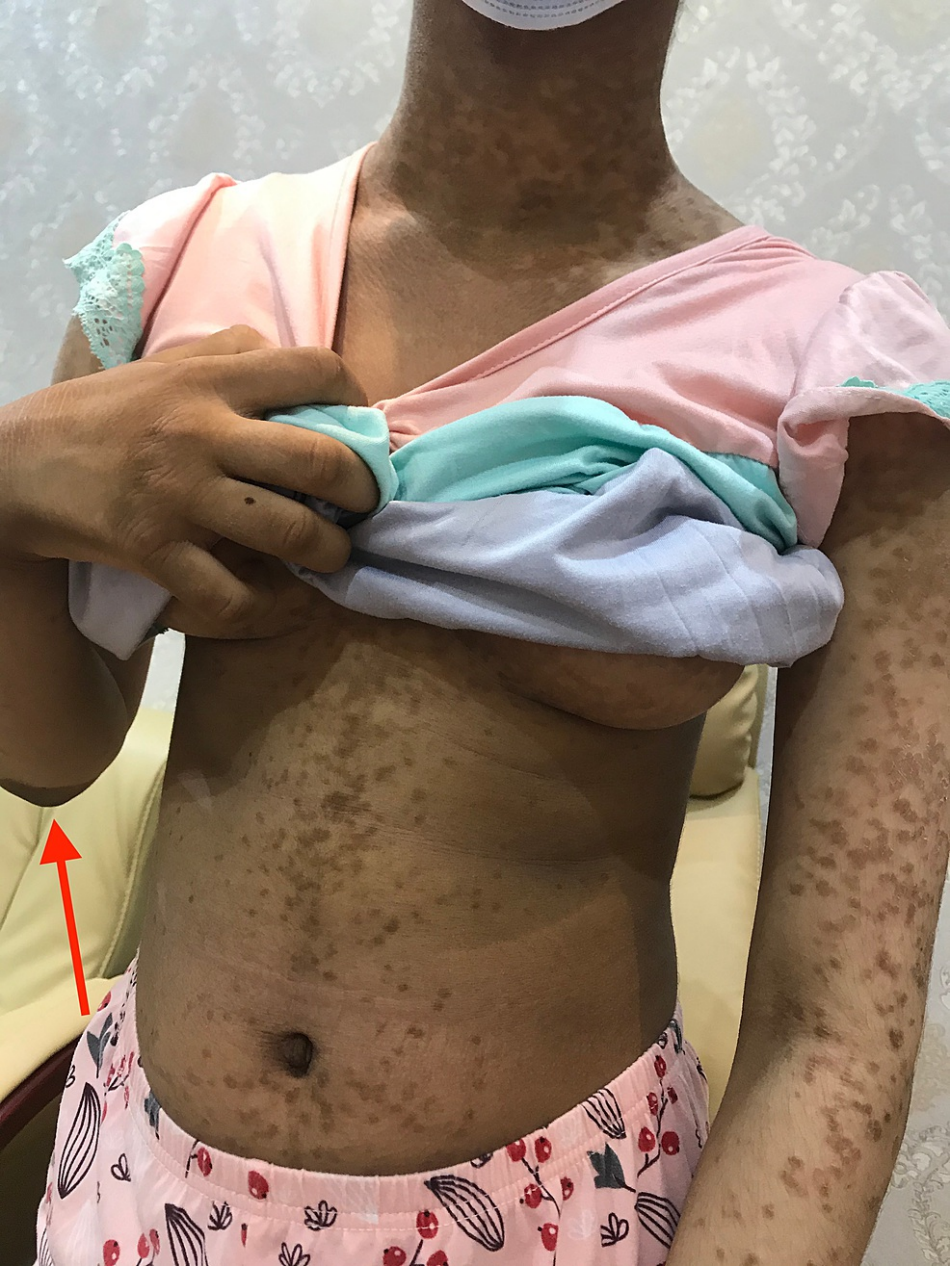
Hyperpigmented reticulated patches of 3-5 mm diameter over the upper trunk of the patient including the neck and arms.

**Figure 3:**
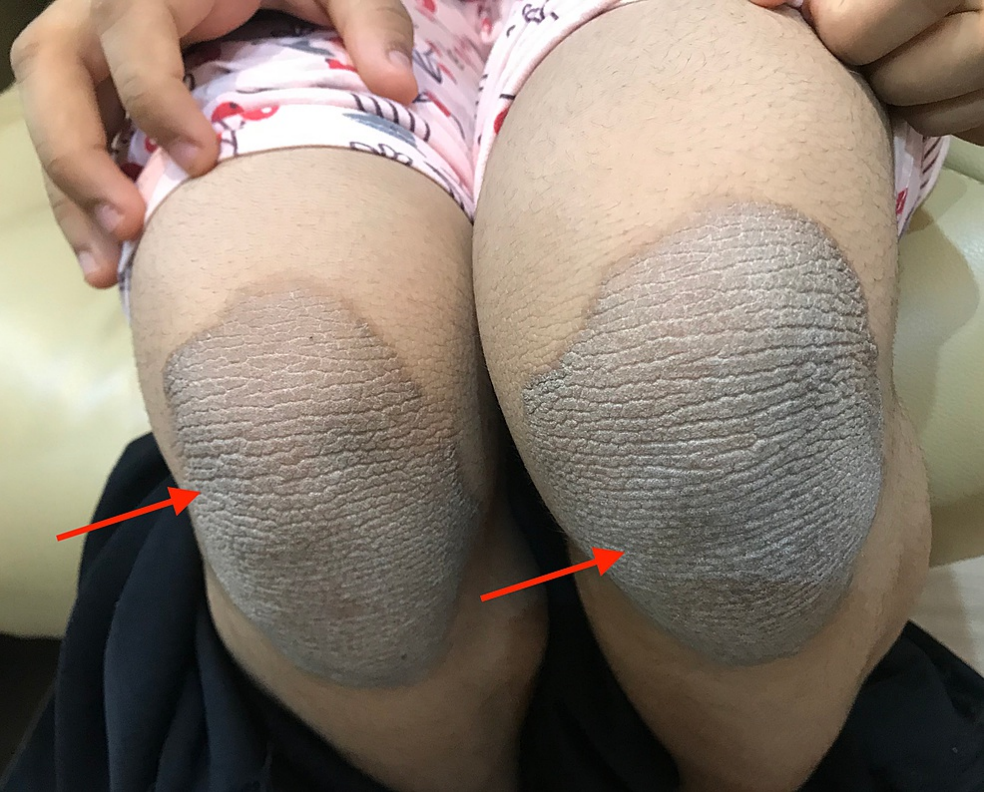
Well-demarcated skin with brownish scaly thin plaques over knees.

A 12-year-old girl, otherwise healthy, presented with a new onset of itchy, persistent, and slowly progressing skin lesions over her body for one year. Systemic examinations, past medical history, drug history, and family background were unremarkable. Skin examination revealed generalized scaly brownish patches, thin papules, and plaques covering her body, including her face, neck, middle of the chest, abdomen, back, upper extremities, elbows, lower extremities, and knees (Figures 1-3). Hair, nails, and mucous membrane examinations were normal.

**Figure 4:**
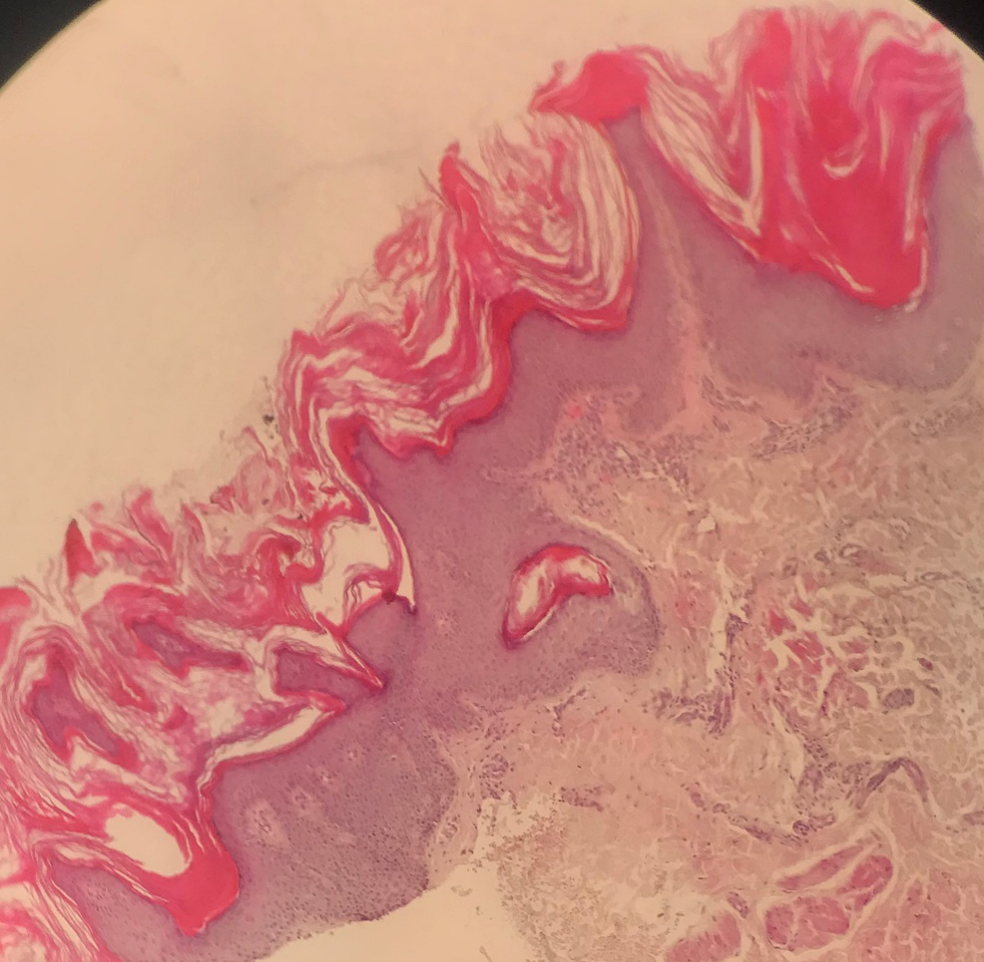
Histopathological features. The epidermis showed papillomatosis, hyperkeratosis, acanthosis, and hypergranulosis. The dermis showed mild perivascular mononuclear cellular infiltrate.

Wood’s lamp examination of her skin lesions was unrevealing. Skin biopsy showed papillomatosis, hyperkeratosis, acanthosis, and hypergranulosis. The dermis showed perivascular inflammatory cellular infiltrate (Figure 4). According to the above clinical and pathological findings, the patient was diagnosed with CARP. She was treated effectively with minocycline, one 85 mg tablet orally for two months, tacrolimus 0.1% ointment twice daily, and selenium sulfide shampoo twice weekly.

## Discussion

CARP was described first in 1927 by two French dermatologists, Gougerot and Carteaud [3]. CARP is a rare chronic disease with exacerbations and remissions, typically affecting young people. The unusual features in our case included the generalized appearance of skin lesions and the appearance of well-defined lesions over the elbows and knees resembling type IV pityriasis rubra pilaris (PRP). However, the histopathological features were typical for CARP. The differential diagnosis in our case included PRP, tinea versicolor, keratosis lichenoides chronica, and symmetrica progressiva. The skin lesions of PRP are not reticulated, and the presence of follicular papules is crucial for PRP [4]. Tinea versicolor skin lesions are not reticulated. The skin lesions of keratosis lichenoides chronica are reticulated and occur around the midline of the body, similar to CARP; however, the lesions of CARP are typically lichenoid [5]. The characteristic histopathological features of CARP, which are club-shaped, bulbous epidermal rete ridges with pigment at their bases (dirty feet), were not seen in our case; however, the other histopathological features in our case were typical for CARP. The most effective treatment for CARP is oral antibiotics (minocycline and doxycycline) [2]. CARP responds to treatment but recurs after discontinuation of the treatment. Table 1 represents our literature review of 21 cases with CARP at different lesion sites for which the majority of cases were localized along the trunk. To our knowledge, no case with generalized lesions, including face, neck, middle of the chest, abdomen, back, upper extremities, elbows, lower extremities, and knees, has been published. Our patient responded well to oral minocycline, a dose of one 85 mg tablet daily, tacrolimus 0.1% ointment twice daily, and selenium sulfide shampoo twice weekly for two months.

**Table 1:** Summary of our literature review of 20 cases with CARP. CARP: confluent and reticulated papillomatosis.

Case	Citation	Year	Gender	Age	Site	Treatment	Outcome
1	Lahouel et al. [6]	2021	Female	20	Trunk, neck, and back	Oral doxycycline 100 mg daily	Two months later, the patient was free of cutaneous lesions. The patient’s skin condition was stable after 1 year of follow-up
2	Lahouel et al. [6]	2021	Male	21	The anterior region of the trunk	Oral doxycycline 100 mg daily	Lesions disappeared completely, with no relapse during the 18-month follow-up period
3	Lahouel et al. [6]	2021	Not found	16	Neck and the trunk	Oral doxycycline 100 mg daily	Complete clearance of lesions in 2 months
4	Amatya et al. [7]	2020	Male	23	Upper chest, back, neck, upper arms, and axillae	Oral minocycline 50 mg twice daily and topical tretinoin 0.05% gel	There was complete resolution of the lesions after two months of treatment and he has remained disease-free for the last six months
5	Lee et al. [8]	2018	female	21	Intermammary region, abdomen, neck, and back (interscapular region)	Oral minocycline 100 mg daily for 8 weeks	Completely cleared with no relapse observed throughout the follow-up until now
6	Lee et al. [8]	2018	Male	17	Abdomen	Oral doxycycline 100 mg 2 times per day for 12 weeks	Completely cleared with no relapse observed throughout the follow-up until now
7	Lee et al. [8]	2018	Female	17	Abdomen, neck, and back	Oral doxycycline 100 mg 2 times per day for 12 weeks	Completely cleared with no relapse observed throughout the follow-up until now
8	Rai and Vishwakarma [9]	2018	Male	23	Chest and anterior part of lower one-third of the neck	Oral minocycline 100 mg daily	The patient is on follow-up
9	Herrera Balam et al. [10]	2018	Female	26	The anterior cervical region, the anterior, posterior thorax, the intermammary area, and the lumbar area	Oral doxycycline 100 mg every 24 hours for 3 weeks and topical retinoic acid in areas affected at night	Three weeks after the start of treatment, the lesions are better observed; however, the remission of the lesion is not reached, and the persistence of lesions in the lumbar area is observed
10	Fukumoto et al. [11]	2017	Female	12	Infra- and intermammary areas and abdomen, neck, axillae, and groin	Oral minocycline 100 mg twice a day	At the end of the total of 10 weeks of oral minocycline therapy, CARP lesions remained completely resolved
11	Hudacek et al. [12]	2012	Female	36	The central aspect of the chest, abdomen, and back	Oral minocycline 100 mg/day for 3 months	Lesions cleared. The patient remained free of lesions at 3 months of follow-up
12	Hudacek et al. [12]	2012	Male	15	Neck, lower abdomen, and lower back	Oral minocycline 100 mg twice daily and topical tazarotene cream 0.05%	The patient reported vast improvement and remained free of rash after 3 months
12	Hudacek et al. [12]	2012	Female	17	Neck, central chest, shoulders, and upper back	Oral minocycline 100 mg/day and topical tazarotene cream 0.1% daily	The lesions improved, and the patient remained free of rash while not receiving any therapy at the 6-month follow-up
14	Hudacek et al. [12]	2012	Female	23	Chest and trunk	Oral minocycline 100 mg twice daily and topical ammonium lactate cream, 12%, twice daily	After 6 weeks of oral and topical therapy, the patient's lesions resolved. Four months later, the patient returned with a recurrence of her lesions. She was again prescribed minocycline 100 mg twice daily for 2 months. Two years later, she again returned with a recurrence, stating that her lesions had been cleared with prior therapy
15	Ferreira et al. [13]	2009	Female	25	The mentalis region, neck, and anterior and posterior regions of the chest	Topical glycolic acid at 12% cream	Improvement was observed 3 months later
16	Ferreira et al. [13]	2009	Female	22	Trunk	Oral 20 mg isotretinoin for 2 months	Improvement within 2 months
17	Ferreira et al. [13]	2009	Male	25	Trunk and anterior and posterior regions	Not taking any treatment	N/A
18	Kim et al. [14]	2009	Male	19	Chest and forehead	Oral minocycline 200 mg/day without any topicals	After 4 weeks, there was complete resolution of the eruption with no relapse for 6 months
19	Lee et al. [15]	2008	Male	18	In both popliteal fossae	Topical methylprednisolone aceponate cream for one week	The lesions faded gradually and cleared within 4 weeks
20	Lee et al. [15]	2008	Male	17	Both elbows, both popliteal fossae, and axillae	Oral minocycline 200 mg every day for 4 weeks and topical ketoconazole cream	This resulted in complete regression of the lesions

## Conclusions

CARP is a rare condition most often occurring in young adults. Classic clinical characteristics include asymptomatic scaly hyperpigmented macules, patches, and papules in a confluent and reticular pattern on the trunk. A generalized form of CARP, as described in our case, was previously reported. CARP is diagnosed based on clinical and histopathological features. Oral antibiotics are the cornerstone of treatment.
